# Dental DNA Mutations Occurring after Death: A Novel Method for Post-Mortem Interval (PMI) Estimation

**DOI:** 10.3390/ijms25168832

**Published:** 2024-08-14

**Authors:** Ilenia Bianchi, Simone Grassi, Eleonora Nardi, Francesca Castiglione, Martina Focardi

**Affiliations:** 1 Laboratory of Personal Identification and Forensic Morphology, Department of Health Sciences, University of Florence, Largo Brambilla 3, 50134 Florence, Italy; simone.grassi@unifi.it (S.G.); martinafocardi@gmail.com (M.F.); 2 Forensic Medical Sciences, Department of Health Science, University of Florence, Largo Brambilla 3, 50134 Florence, Italy; 3 Section of Anatomic Pathology, Department of Health Sciences, University of Florence, Careggi University Hospital, Largo Brambilla, 50134 Florence, Italy; eleonora.nardi@unifi.it (E.N.); francescacastiglione@gmail.com (F.C.)

**Keywords:** post-mortem interval (PMI), accumulated degree days (ADDs), next-generation sequencing (NGS), teeth, dental DNA, forensic odontology, molecular biology

## Abstract

Post-mortem interval (PMI) estimation remains one of the major challenges in forensic practice, especially for late PMIs beyond 7–10 days after the death of the subject. In 2022, an innovative method to investigate the occurrence of mutations induced by the death of a subject in the DNA of post-mortem dental pulps at different PMIs was developed, applying a next-generation sequencing (NGS) analysis. The present study aims to apply the same method of analysis to a small sample of teeth belonging to the same subject and analyzed at different PMIs/accumulated degree days (ADDs), and of teeth extracted from different subjects but analyzed at the same PMI/ADD to verify the repeatability of the results obtained in relation to the time elapsed since death. A total of 10 teeth were collected from 6 patients (3 males and 3 females) with PMI varying from 8 to 35 days, and ADD from 157.4 to 753.8. We found 1754 mutations in 56 genes, with more than 700 mutations having a prevalence > 5% and more than 300 variants considered of interest for the purposes of the study. Mutations that were not present at lower PMIs but manifested in later PMIs in pulps belonging to the same subject demonstrate that they can only have been acquired by the subject after death and according to the time elapsed since death. In total, 67 somatic mutations in 29 out of the 56 genes of the used panel occurred in a fashion that allows an association with specific PMI/ADD ranges (within 8 days, between 17 and 28, and beyond 30 days after death). The results suggest that temperature and humidity could influence the rate of DNA degeneration in dental pulps, thus PMI should be estimated in ADD more than days. The preliminary validation supports the hypothesis that the innovative method could be a useful tool for estimating the post-mortem interval even beyond the first week after death, but further analyses are needed to customize a specific genetic panel for forensic investigations and verify the influence of degenerative processes of soft tissues surrounding dental elements on DNA degeneration of pulps.

## 1. Introduction

The estimation of the post-mortem interval (PMI) remains one of the major challenges in forensic practice, especially for late PMIs beyond 7–10 days after the death of the subject [1,2,3]. Although the common and reliable application of the “compound method” is based on algorithms combining different parameters (in particular, body and environmental temperature), this method can no longer be used beyond 72 h after the subject’s death [4,5]. Most available tools for late PMI estimates (beyond the first week after death) are plagued by significant measurement errors that often have questionable legal meaning [6,7,8].

For this reason, the current literature is strongly interested in the search for novel indicators and methods that can be applied in the estimation of late PMIs with significant reliability, and, in particular, molecular analyses based on the degradation patterns of proteins, DNA, and RNA yielded promising results for forensic purposes [9,10,11,12,13,14,15,16,17]. Genetic analysis of DNA/RNA integrity after death highlighted a progressive correlation of the increase in nucleic acid degradation products with late PMI, even up to 18 months PM [18,19,20,21,22,23,24]. However, nucleic acids are extremely susceptible to external agents that can alter and damage their structures, influencing the speed and degree of post-mortem degradation [18]. In particular, water and temperature significantly influence RNA and DNA conformations (chromatin, tertiary and quaternary structure, helical structure of double-stranded, etc.), causing the opening and denaturation of nucleic acid base pairs by breaking or creating inter-/intra-hydrogen bonds and modifying molecular affinity [25,26,27,28].

Teeth are known for their high resistance to the most extreme temperature variations thanks to the presence of their external enamel, which protects the nucleic acids of the dental pulp, which is rich in cells and blood and is more stable and persistent than many other tissues of the organism [29,30,31]. Furthermore, various dental DNA extraction techniques have been developed that allow genetic analyses to be repeated reliably even on samples of very small quantities [32,33,34,35].

Few studies have focused on the analysis of the integrity of post-mortem DNA of dental pulps and, in particular, on the estimation of the progressive reduction of DNA concentrations related to the time elapsed since death and on the amount of nucleic acid degradation products; nevertheless, these studies are still preliminary and require further validation of the results [36,37]. In particular, it has been highlighted that PM degradation of RNA is much more variable and rapid than that of DNA since RNA, unlike DNA, is already degraded in vivo by ribonucleases ubiquitously present in all tissues [37]. On the contrary, the reduction in the integrity of the nucleic chain (RIN) and in the quantity of DNA can be so slow, up to a year and a half after death, that it can’t be correlated unambiguously with late PMIs [36].

To overcome these limitations, in 2022, we developed an innovative method to investigate the changes and alterations induced by the death of a subject in the DNA of post-mortem dental pulps at different PMIs, applying a next-generation sequencing (NGS) analysis [38]. Preliminary results highlighted the occurrence of somatic mutations caused by cellular necrosis and related to the post-mortem interval calculated both in days and in accumulated degree days (ADDs). The study confirmed that it is possible to detect a number of post-mortem mutations in some target genes of forensic interest in dental pulp cells up to at least 34 days after death. The occurrence or the absence of mutations (alone or as a cluster of the same gene) provided three possible interpretations of the post-mortem interval estimate; some mutations occurred only at specific PMI/ADD or in specific PMI/ADD intervals, but others occurred at every PMI/ADD except for some specific intervals.

According to the literature that investigates factors influencing the rate of decomposition of the different parts of a corpse (TBS, the total body score), post-mortem transformations are better modeled as dependent on accumulated temperature and not just on time elapsed since death [39,40,41]. Therefore, the PMI estimation is more reliable using the accumulated degree-days (ADDs), which consider both time and temperature as the sum of the daily average ambient temperatures between the time of the death and the time of the corpse rescue. Furthermore, ADDs have been proven to improve not only PMI estimates based on the evaluation of body decomposition but are widely applied in entomological methods [42] and also investigated for molecular techniques, considering that temperature considerably influences nucleic acid degeneration [43].

The pilot study [38] found encouraging results in applying NGS analysis on dental DNA for PMI of several days after death but had some limitations. In the preliminary phase, different PMI/ADD corresponded to different cases (teeth/patients); therefore, the sample did not allow a validation of the results in different subjects analyzed at the same PMI nor of the progressive appearance of different mutations in the same subject but analyzed at different PMIs.

This study aims to apply the same method of analysis to a small sample of teeth belonging to the same subject and analyzed at different PMI/ADDs, and of teeth extracted from different subjects but analyzed at the same PMI/ADD. The scope is to verify the repeatability of the results obtained in relation to the time elapsed since death and to exclude the presence of genetic predispositions of the subject to the expression of specific mutations.

## 2. Results

A total of 10 teeth extracted from six patients (three males and three females) were eligible for this study. Samples ID 3–4–7–9 and 2–10 belonged to the same subjects, A and B respectively; the other samples belonged to different subjects. The PMI varied from 8 to 35 days, while the ADD varied from 157.4 to 753.8 (Table 1).

We found 1754 mutations in 56 genes, with more than 700 mutations having a prevalence > 5%. More than 300 variants of interest were considered for the purposes of the study, occurring in an irregular fashion at different PMI/ADDs in the same subject or confirming the presence of the same mutations in different subjects according to the same PMI or ADD (Supplementary Material).

In particular, Table 2 and Table 3 highlight some significant mutations found in 4 teeth extracted from subject A (sample 3–4–7–9) and in 2 teeth from subject B (sample 2–10), and respectively analyzed at different PMIs. The NGS analysis detected the occurrence of different PMI/ADD mutation variants, even in pulps from the same subject. Mutation c.*1363G>A of the *ABL1* gene in subject A (Table 2) occurs only up to PMI 23 and then disappears for the latest PMI. In contrast, the *APC* gene shows a pattern of mutations (*c.1162G>A*, *c.1749G>A*, *c.1840G>T*, *c.2176T>C*, *407C>T*) that manifested only at the 30-day PMI (Table 2), such as the *ROS1* gene, variants of which occur only at PMI 28.

Similarly, subject B (Table 3) showed the occurrence of mutation variants just at time 17 after death (i.e., ATK3 c.*3694G>A, *APC c.7504G>A*); on the contrary, some mutations could be found just at PMI 30 (i.e., *ABRAXAS1 c. 1117G>A*, *ADGRA2 c.3660C>T*).

Moreover, Table 4 reports the mutations found in teeth extracted from different subjects and analyzed at the same PMI/ADDs. Column 2 reports the results obtained from a sample analyzed at PMI 8 in the 2022 study [34] and compared with sample 8 of this study at the same PMI (Column 1). Columns 3 to 11 report the results obtained from the samples analyzed in this study. We could detect some mutations occurring only at specific PMI/ADD or at specific ranges of PMIs/ADD. In particular, mutation variant *c.7274G>A* of gene *ATR* can be found solely in early PMIs (8–16 days), whilst mutation *c.135C>T* of gene *AMER1* only for PMIs longer than 30 days, both confirming the occurrence in PM pulps from different subjects at the same PMIs. Some other mutations can be found only at specific PMI ranges, e.g., *c.4204C > T* of gene *ANKRD26* or *c.3068-574C > A* of gene *ALK* between 16 and 28 PM. The latter case manifested in both subjects analyzed at PMI 16, but up to 30 days only in one subject (Columns 8 and 9).

## 3. Discussion

The main field of research in forensic odontology is well-known as personal identification and age estimation [44,45,46,47]. Equally, it is well known that teeth are a reliable source of DNA for identification purposes, as the pulp is protected from damaging factors and it is rich in DNA, more stable than in other tissues, or, in cases of widely destroyed corpses, sometimes the sole source for genetic analyses [29,30,31,32,33,34,35,47].

According to the few available studies, it emerges that molecular investigations of dental pulp nucleic acids can be applied in correlating post-mortem DNA alterations with the time elapsed since death, but DNA degradation yields long plateau periods which do not allow the use of these methods for estimating late PMIs (more than 7–10 days after death) [36,37,43].

Thus, in 2022, we developed an innovative method to investigate the occurrence of somatic mutations caused by cellular necrosis induced by the death of a subject in the DNA of post-mortem dental pulps, applying a next-generation sequencing (NGS) analysis [38].

The study confirmed that it is possible to detect some post-mortem mutations in target genes eligible for the purpose of the PMI estimation, but we applied a specific panel for detecting somatic mutations due to oncological diseases affecting different tissues since no previous research was conducted on a specific genetic panel to detect post-mortem DNA damage. Furthermore, the sample was too small to verify the repeatability of the results obtained at the same PMI and, therefore, to validate the correlation of the occurred mutations with PMI/ADDs. Another limitation was related to the unknown mechanism of mutations after the death of the dental pulp, since no previous literature is available. The oncopanel that we used discards multiple polymorphisms [48,49,50], and the analyzed pulps were vital, not targeted by any oncological diseases, and coming from healthy patients with an unremarkable medical history, so detected mutations could be attributed solely to the post-mortem DNA changes. Nevertheless, the collected teeth belonged to different subjects, so it was not possible to verify the existence of a genetic predisposition of the subject in manifesting some mutations compared to others in relation to the PMI.

We performed targeted NGS on 10 sound teeth extracted from healthy patients with the aim of better understanding the feasibility of the innovative method for the PMI estimation developed in 2022 [38]. According to the pilot study, we considered both PMI and ADD, storing teeth at room temperature to verify the susceptibility of the nucleic acids of the pulp to different temperature and humidity conditions (ADDs) as well as the time elapsed since death calculated in days [51]. Mutation variants with an allelic frequency of <5% were excluded from the study to minimize the risk of error.

In this study, we selected six teeth from two different subjects (in particular, four teeth from subject A (Table 2), and two teeth from subject B (Table 3)), which were analyzed at progressive PMIs. The NGS found the occurrence of some different PMI/ADD mutation variants even in pulps from the same subject (Table 2 and Table 3). In particular, for subject A, the DNA alterations at PMI 16, 23, 28, and 30 were analyzed, respectively. Mutation c.*1363G>A of the *ABL1* gene in subject A (Table 2) occurs only up to PMI 23 and then disappears for the latest PMI. In contrast, the *APC* gene shows a pattern of mutations (*c.1162G>A*, *c.1749G>A*, *c.1840G>T*, *c.2176T>C*, *407C>T*) that manifested only at the 30-day PMI (Table 2), such as *ROS1* gene, variants of which occur only at PMI 28. Similarly, subject B (Table 3) showed the occurrence of mutation variants just at time 17 after death (i.e., ATK3 c.*3694G>A, *APC c.7504G>A*); on the contrary, some mutations could be found just at PMI 30 (i.e., *ABRAXAS1 c. 1117G>A*, *ADGRA2 c.3660C>T*). Since the analyzed pulps belonged to the same subjects, the variable that may have determined the appearance or the disappearance of one mutation variant compared to another can be attributed solely to the time elapsed since death. Furthermore, some mutations of the same genes were not present at earlier PMIs but just manifested in later PMIs, e.g., *APC* (variants *c.5034G>A*, *c.5268T>G*, *c.5465T>A*, and *c.5880G>A* appear only up to 28 days; variants *c.1162G>A*, *c.1749G>T*, *c.2176T>C*, and *c.407C>T* appear only at 30 days (Table 2)) or *EGFR* (e.g., variants *c.274T>C* or *c.2868T>C* (Table 2)), demonstrating that they can only have been acquired after death by the subject. This finding also suggests that a single gene could be applied in investigating different PMIs (early and late), customizing the panel to be applied for forensic purposes. The selection and reduction of the number of genes to be analyzed for PMI estimation allows the high costs and analysis times to be significantly reduced.

The comparison between the results obtained in samples from different subjects analyzed at the same PMI, found 67 somatic mutations in 29 out of the 56 genes of the used panel (Table 4). The other genes and mutation variants were considered of scarce interest because they did not occur in a fashion that allows an association with specific PMI/ADD. For instance, two mutations (c.*35C>T, and c.*36A>C) of *CSF1R* were found at every PMI/ADD, with prevalence values ranging from 50 to 100%, confirming the results found in the pilot study of 2022 [38].

The selected variants (Table 4) were considered of interest for the purposes of the study, occurring in an irregular fashion that allows attribution to specific PMI/ADD ranges. In detail, some mutations, such as variant *c.7274G>A* of gene *ATR*, can be found solely in early PMIs (8–16 days), while mutation *c.135C>T* of gene *AMER1* is only for PMIs longer than 30 days, both confirming the occurrence in PM pulps from different subjects at the same PMIs. Some other mutations can be found only at specific intermediate PMI ranges, e.g., *c.4204C>T* of gene *ANKRD26* or *c.5624-1340T>C* of gene *ROS1*. These findings seem to indicate that the method could be a useful tool for estimating the post-mortem interval in specific time ranges occurring after death, which could be set within 8 days, between 17 and 28 days, and beyond 30 days post-mortem and, therefore, also useful for late PMIs for which no other reliable methods are available.

However, the preliminary validation of the results at the same PMI highlights a correspondence of some mutation variants at PMI 8, 16, and 35 but not at PMI 30 (Columns 8 and 9 of Table 4). For example, mutation *c.3068-574C>A* of gene *ALK* occurred between PMI 16 and 30, but, in the case of PMI 16, it was found for both analyzed subjects, while at PMI 30 only in one subject (Column 9, Table 4). In particular, sample 4 at 30-days PMI (Table 1) seems to be endowed with a better concordance with pulps analyzed at PMI 35 (samples 5–6, Table 1) rather than with the other control at 30 days PM (sample 2). In detail, the recorded ADD value of the last three samples (2, 5, and 6) of Table 4 (one at 30 and two at 35 days after death) is similar (about 730 °C), while the ADD registered for sample 4, analyzed at 30 days after death, is about 580 °C, thus suggesting that temperature and humidity could infer the rate of DNA degeneration in the dental pulp and that the more reliable measurement unit for estimating the post-mortem interval is ADD more so than days. This is a novel finding that needs to be verified by further research on larger samples of different subjects analyzed at different established PMIs and ADDs.

According to the findings from the same subjects at increasing PMIs (Table 2 and Table 3), mutation occurrence increases according to time elapsed since death, confirming the preliminary results of the pilot study of 2022 [38]. Furthermore, most of the genetic variants that occur in post-mortal pulps are substitutions of nucleotide bases (Table 4). This mechanism could be explained with a different passive affinity between the increase in free concentrations of nucleotide bases and the progressively damaged DNA [41] in necrotic dental pulps. Further research is needed to investigate the mechanism of the PM dental pulp mutations occurrence.

In this study, mainly premolars obtained from the same subjects who underwent multiple extractions for orthodontic reasons were analyzed. For the validation phase, it was necessary to obtain multiple dental elements from the same subject at different PMIs, and, therefore, all types of teeth were included in the sample collection, differently from the pilot study of 2022 [38], for which only molars were selected as preferable dental elements for forensic purposes since the extraction of posterior teeth is more conservative for corpses under judicial autopsy. Further studies are needed to verify if tooth type could influence the occurrence of mutations. The use of teeth extracted from living subjects allows us to know the exact time of death. Nevertheless, further studies are needed to investigate the influence of cadaveric processes of soft tissues surrounding dental elements (e.g., the periodontal ligament, gingival mucosa, tongue, etc.) as well as different preservation conditions of corpses (e.g., in water or remains buried at different depths and soil characteristics) on the degradation of dental pulp nucleic acids [52,53]. Furthermore, only pulp analysis was selected for this method. Due to the difficulty of collecting an adequate number of samples and the slight quantity of dental pulps, it could be interesting to apply the method also to the DNA of dental root hard tissues [54], to determine whether the presence of mutations is influenced by the type of dental tissue and to improve the number of analyses that can be performed on the same subject.

In our opinion, the preliminary validation of results supports the hypothesis that the innovative method could be a useful tool for estimating the post-mortem interval even beyond the first week after death, and that it is crucial to deepen research to customize a specific genetic panel for forensic investigations and test different dental tissues (soft and mineralized) in different conditions of humidity, temperature, burial, and cadaveric degeneration.

## 4. Materials and Methods

### 4.1. Data Loggers, Genetic Kits, and Chemical Reagents

According to the protocol developed in the pilot study of 2022 [38]:

- IButton DS1923 Hygrochron Temperature/Humidity Data Logger, iButtonLink Technology, LLC, Whitewater, WI, USA
- Express-Thermo, Eclo Solutions, 2014 version, Leiria, Portugal
- SecurBiop^®^ e FORMALeasy^®^, Techingreen Srl, Teramo, Italy; 4% buffered formaldehyde
- MagCore genomic DNA FFPE onestep, Diatechlabline pharmacogenetics, Jesi, Italy
- Myriapod NGS-L T 56G Oncopanel Kit
- q-PCR, Rotor-gene, Qiagen, Hilden, Germany
- Qubit dsDNA HS Assay kit, Invitrogen by Thermo Fisher Scientific, Midland, Canada
- Ion One Touch 2 instrument, Life Technologies, Grand Island, The Netherlands
- Dynabeats MyOne Streptavidin C1 Beads, Invitrogen by Thermo Fisher Scientific, Midland, ON, Canada
- Ion S5 System, Ion Torrent platform, Invitrogen by Thermo Fisher Scientific, Midland, Canada
- Myriapod NGS Data Analysis Software, Myriapod NGS Analysis software e Myriapod workstation NGS, Diatech Pharmacogenetics srl, Jesi, Italy

### 4.2. Sample Collection and Storage

The study considered human teeth extracted from healthy living patients for clinical reasons at the Dental Department of Careggi University Hospital, in Florence (Italy), and obtained the approval from the local ethics committee (n. 15208/2020). Consent for research and publication was obtained in all the cases, and data were processed in compliance with the European Union law (GDPR).

We used teeth from living people rather than from real autopsies, to have certain PMIs, as the interruption of blood supply to the dental pulp at the moment of tooth avulsion is comparable to the moment of the subject’s death, and the PMI can be considered as the time elapsed from the dental extraction.

Inclusion criteria were:

- Patients who underwent multiple tooth extractions for orthodontic reasons;
- Unremarkable medical history of patients;
- Permanent, completely mineralized, sound, and unrestored incisors, canines, premolars, and molars. All teeth were considered eligible to collect multiple elements, as possible, from the same subject.
- Normal response to pulp vitality tests (i.e., thermal tests)

Exclusion criteria were:

- Decayed, fractured, or damaged teeth.
- Teeth treated with conservative, endodontic, or prosthetic therapies.
- Non-vital teeth or abnormal results of vital pulpal tests.
- Medical history positive for relevant diseases (e.g., cancer, diabetes) or chronic drug treatments.

An eligible sample composed of 10 teeth extracted from 6 different patients was then selected for the study. Sex, age, tooth position, and date of the avulsion were registered for each patient.

Immediately after the avulsion, teeth were stored at non-standardized temperatures, and conditions to simulate real corpse conservation and accumulated degree-days (ADDs) were registered using the IButton DS1923 Hygrochron Temperature/Humidity Data Logger (iButtonLink Technology, LLC, Whitewater, WI, USA), set up at intervals of 15 min according to the method developed in 2022 [38]. The data logger remained active throughout the period elapsed from the tooth extraction up to the freezing of the dental pulp [at −20 °C] for the NGS analysis, considered the PMI. ADD reports for each tooth were obtained using the appropriate Software Express-Thermo (Eclo Solutions, 2014 version, Leiria, Portugal).

According to the pilot study [38], ADDs were calculated as: *(T maximum + T minimum)/2—T threshold*, where T maximum indicates maximum temperature reached during the day, T minimum, the minimum temperature reached during the day, and T threshold, which represents the value above which the phenomenon examined takes place that, in the case of nucleic acid degradation, was considered 0 °C, since sample freezing inhibits the process [55].

### 4.3. Dental Pulp Extraction

The extraction of pulp tissue from each tooth was performed at a set PMI/ADD according to the final observation purpose. Teeth extracted from the same subject were frozen at −20 °C once they reached different ADD sets in order to evaluate the presence of genetic predispositions of the subject to the expression of specific mutations; teeth obtained from different subjects were frozen at the same PMI/ADD to verify the repeatability of the results obtained in relation to the time elapsed since death.

The pulp extraction technique followed the same protocol proposed in the pilot study of 2022 [38]. A sulcus depth of about 1 mm at the apical level and 2 mm at the coronal level of dental enamel was executed with a finishing bur, mounted on a turbine, with abundant irrigation, to guide the succeeding tooth fragmentation with a hammer and a thin lever. Then, pulp fragments were removed using tweezers, endodontic files, or small excavators and fixed with 4% buffered formaldehyde (60 mL Securbiop biopsy container, SecurBiop^®^ e FORMALeasy^®^, Techingreen Srl, Teramo, Italy) for up to 12 h at low temperatures, according to the DNA extraction and analysis procedures described by Berrino et al. [56,57].

### 4.4. DNA Extraction and NGS Analysis

DNA extraction and NGS analysis followed the same method described in the pilot study of 2022 [38], and the same “Myriapod NGS-L T 56G Oncopanel Kit” (Diatechlabline pharmacogenetics, Jesi, Italy) was applied for the quantity and quality (fragmentation) evaluation of the gDNA through quantitative polymerase chain reaction (q-PCR, Rotor-gene, Qiagen, Hilden, Germany).

Only the sequences that had a quality index (PHRED score) of at least Q > or = a 20 were considered, while the minimum coverage considered in each sample was 100 reads per amplicon. The non-synonymous mutations were selected using on-line genetic databases (such as COSMIC, LOVD, PUBMED, Clinvar, last accessed on 10 May 2022) for the variants already described, while for the genetic polymorphisms (single-nucleotide polymorphism, SNP), dbSNP and 1000 Genome. For the mutations not yet described, the pathogenetic prediction “tools” of the variant were used, such as WebAnnover and Provean (SIFT and Pholyfen).

## 5. Conclusions

The study confirms that post-mortem mutations occur in dental pulp DNA and may be correlated with the time elapsed since death. Dental pulp DNA seems to be endowed with a genetic predisposition for some mutation variants occurring after death, but some specific genes yielded promising results in correlation with time elapsed since death and, specifically, for late PMI ranges (up to 30 days PM). The pulp DNA analysis method using NGS appears to be repeatable, but it is necessary to customize a specific genetic panel for forensic investigations that includes all the genes and variants of interest for estimating the post-mortal interval, thus reducing costs and time of analyses.

It is necessary to delve deeper and understand the mechanism that determines the occurrence of PM mutations to better interpret results.

The preliminary validation of results suggests that PMI estimation could be more reliable by considering ADDs rather than days, since temperature and humidity can influence the rate of post-mortem mutation occurrence. Further studies are necessary to validate the method on a large sample, including pulps from different subjects analyzed both at the same and different PMIs and ADDs, and different post-mortem conditions, such as water, burial, temperature, and PM transformation degrees of soft tissues surrounding dental elements.

## Figures and Tables

**Table 1:** Data set of eligible samples. Tooth position, PMI, and ADD.

CASE	TOOTH *	PMI (Days)	ADD (°C)
8	LLTM	8	157.4
1	URCI	16	388.2
7	LRFP	16	369.2
10	LRSP	17	440.9
3	ULFP	23	551.2
9	URFP	28	678.3
2	URSP	30	738.1
4	LLFP	30	581.5
5	LLSP	35	753.8
6	LRSP	35	753.8

* URCI: upper right central incisor; URSP: upper right second premolar; ULFP: upper left first premolar; LLFP: lower left second premolar; LLSP: lower left second premolar; LRSP: lower right second premolar; LRFP: lower right first premolar; LLTM: lower left third molar; URFP: upper right first premolar.

**Table 2:** Some significant mutations found in subject A: 4 premolar teeth analyzed at different PMI/ADDs, with mutations found at specific PMIs or PMI/ADD ranges. Case 7—LRFP at PMI 16; case 3: ULFP at PMI 23; case 9: URFP at PMI 28; case 4: LLFP at PMI 30 (Supplementary Material for the complete mutations set). Coloured boxes represent the occurrence of mutations; clear boxes represent the absence of mutations.

PMI (Days)		**16**	**23**	**28**	**30**
ADD (°C)		**369.2**	**551.2**	**678.3**	**581.5**
GENES	**MUTATIONS**				
ABL1	c.*1363G>A				
APC	c.1162G>A				
	c.1749G>A				
	c.1840G>T				
	c.2176T>C				
	c.407C>T				
	c.5034G>A				
	c.5268T>G				
	c.5465T>A				
	c.5880G>A				
BRAF	c.*1363G>A				
	c.*1483A>G				
	c.*813T>C				
	c.*922G>C				
	c.3324A>G				
	c.5094C>T				
	c.6555C>T				
	c.75+60C>T				
EGFR	c.1839C>T c.1880+55C>T				
	c.1881-1267C>A				
	c.1881-1585A>G				
	c.1881-2094A>G				
	c.1881-404T>G				
	c.1881-721G>A				
	c.1887T>A				
	c.2283+125T>C				
	c.2283+1296C>T				
	c.2283+1901G>A				
	c.2283+2225C>T				
	c.2283+269T>C				
	c.2283+2994A>G				
	c.274T>C				
	c.2868T>C				
	c.2983C>T				
	c.3014G>A				
	c.3127T>C				
	c.489C>T				
	c.563G>A				
	c.622-248G>A				
	c.686T>C				
	c.738C>T				
ESR1	c.1096+21499_1096+21500				
	c.1236-18935G>A				
ETV1	c.182-3581A>G				
	c.182-3990C>T				
	c.182-4016G>C				
	c.182-4229 182-4228delinsGC				
	c.235+113A>G				
	c.235+11542C>T				
GRM3	c.879C>T				
H3F3C	c.*2T>G				
KRAS	c.*512T>C				
	c.-12+2376_-12+2391dup				
	c.2049A>G				
	c.2225G>C				
	c.2547=				
	c.2629A>T				
	c.2718A>G				
	c.387G>A				
	c.3906T>C				
	c.451-5617G>A				
	c.451-7404A>C				
PDGFRA	c.1701 A>G				
	c.1787-269G>A				
	c.3222T>C				
ROS1	c.5231-1578A>G				
	c.5231-191C>T				
	c.5231-2516A>G				
	c.5231-2735T>C				
	c.5231-3291C>T				
	c.5231-3311T>C				
	c.5231-415C>T				
	c.5231-735T>C				
	c.5348+1319G>A				
	c.5348+361T>A				
	c.5624-1461G>T				
	c.5624-876A>G				
	c.5759+351 del				
	c.5759+468A>C				
	c.5759+603A>C				
	c.5760-137G>A				
	c.5760-281C>T				
	c.5760-53G>T				
	c.5922+434G>C				

**Table 3:** Some significant mutations found in subject B, two premolar teeth analyzed at different PMI/ADDs, with mutations found at specific PMI/ADDs. Case 10: LRSP at PMI 17; case 2: URSP at PMI 30 (Supplementary Material for the complete mutations set). Coloured boxes represent the occurrence of mutations; clear boxes represent the absence of mutations.

PMI (Days)		**17**	**30**
ADD (°C)		**440.9**	**738.1**
GENES	**MUTATIONS**		
ABRAXAS1	c.1117G>A		
ADGRA2	c.3660C>T		
AKT2	c.-84-5059-84-5052		
AKT3	c.*3694G>A		
	c.*3731C>T		
APC	c.7504G>A		
BRCA1	c.-19-216A>G		
	c.2311T>C		
	c.2612C>T		
	c.3119G>A		
	c.3548A>G		
	c.4308T>C		
	c.4837A>G		
	c.5075-237C>A		
CCND1	c.723+571		
	c.723G>A		
CCND2	c.493C>T		
CCNE1	c.1215C>T		
	c.399T>C		
ETV6	c.463+7490C>A		
	c.463+7929G>A		
	c.463+848G>C		
	c.464-6175G>T		
	c.464-6269C>T		
RPS6KB2	c.1259C>T		
	c.807C>T		
RPTOR	c.1269C>T		
TNFRSF14	c.50A>G		
TP53	c.1100+154C>G		
ZFHX3	c.1212C>T		
	c.1282A>C		
	c.1602C>T		

**Table 4:** Mutations found in teeth extracted from different subjects and analyzed at same PMI/ADDs. Column 2 reports the results obtained from a sample analyzed at PMI 8 in the 2022 pilot study and compared with the results obtained from sample 8 (Column 1) from this study. Columns 3 to 11 compare the samples analyzed in this study. Coloured boxes represent the occurrence of mutations; clear boxes represent the absence of mutations.

**PMI (Days)**		**8**	**8**	**16**	**16**	**17**	**23**	**28**	**30**	**30**	**35**	**35**
**ADD (°C)**		**157.4**	**183.1**	**369.2**	**388.2**	**440.9**	**551.2**	**678.3**	**581.5**	**738.1**	**753.8**	**753.8**
**GENES**	**MUTATIONS**											
ABRAXAS1	c.1117G>A											
AKT3	c.*3909T>C											
ALK	c.3067+365C>T											
	c.3068-415G C											
	c.3068-574C>A											
	c.3359+24G>C											
	c.3375C>A											
	c.4338C>T											
	c.4472A>G											
AMER1	c.135C>T											
ANKRD26	c.4204C>T											
ATR	c.7274G>A											
AXIN1	c.1284G>A											
	c.1549G>A											
	c.1827T>C											
BAP1	c.501G>A											
BCL2L11	c.394+3056G>C											
	c.394+3849C>T											
BCL2L2	c.128A>G											
BCL6	c.492G>T											
BLM	c.1046G>A											
	c.3102G>A											
	c.3531C>A											
	c.3945C T											
BMPR1A	c.1140C>T											
BRAF	c.1140+757A>G											
	c.1141-1305T>C											
BRCA1	c.134+1967T>C											
	c.134+2983_134+3004delinsAAACCCCTACTGATGAA											
	c.-19-115T>C											
	c.-19-216A>G											
	c.3113A>G											
	c.3119G>A											
	c.3548A>G											
	c.4308T>C											
	c.4837A>G											
	c.5075-237C>A											
CCND1	c.723+571											
	c.723G>A											
EGFR	c.1881-721G>A											
	c.1881-781C>T											
	c.747+548C>T											
	c.748-235C>T											
	c.89-25201A>G											
EML4	c.*1941A>G											
ERBB2	c.2086-29G>A											
	c.2273delA											
FAT1	c.8523C>T											
FGFR3	c.2393delC											
	c.840C>T											
	c.843A>C											
FLI1	c.203G>T											
	c.942C>G											
HNF1A	c.1375C>T											
	c.1460G>A											
KAT6A	c.401T>C											
	c.4455C>T											
KDM5A	c.2594T>C											
	c.4149C>T											
PDGFRA	c.2003-11delT											
PTEN	c.802-18delC											
	c.802-19dupT											
	c.802-23C>T											
	c.802-29C>T											
	c.80-3023C>T											
ROS1	c.5624-1340T>C											
TNFRSF14	c.50A>G											

## Data Availability

No new data were created or analyzed in this study. Data sharing is not applicable to this article.

## Supplementary Materials

The following supporting information can be downloaded at: https://www.mdpi.com/article/10.3390/ijms25168832/s1.
