# Supplementary material for: Dental DNA Mutations Occurring after Death: A Novel Method for Post-Mortem Interval (PMI) Estimation
Source: Int J Mol Sci. 2024 Aug 14;25(16):8832. doi: 10.3390/ijms25168832 (PMC11354992; doi:10.3390/ijms25168832)
Supplement: Supplementary file 1 [file ijms-25-08832-s001.zip › ijms-3108740-supplementary.pdf]

**Supplementary Material**

**Table S1.** Complete mutations set found in subject A: 4 premolar teeth analyzed at different PMI/ADDs, with mutations found at specific PMIs or PMI/ADD ranges. Case 7—LRFP at PMI 16; case 3: ULFP at PMI 23; case 9: URFP at PMI 28; case 4: LLFP at PMI 30. Coloured boxes represent the occurrence of mutations; clear boxes represent the absence of mutations.

| **PMI (Days)** | | **16** | **23** | **28** | **30** |
|---|---|---|---|---|---|
| **ADD (°C)** | | **369.2** | **551.2** | **678.3** | **581.5** |
| **GENES** | **MUTATIONS** | | | | |
| ABL1 | c.*1363G>A | ■ | ■ | | |
| AKT3 | c.*3909T>C | ■ | ■ | | |
| ALK | c.4338C>T | ■ | ■ | ■ | |
| ANKRD26 | c.-140C>G | ■ | ■ | | |
| | c.212-1110A>G | | | | ■ |
| APC | c.1162G>A | | | | ■ |
| | c.1749G>A | | | | ■ |
| | c.1840G>T | | | | ■ |
| | c.2176T>C | | | | ■ |
| | c.407C>T | | | | ■ |
| | c.5034G>A | ■ | ■ | ■ | |
| | c.5268T>G | ■ | ■ | ■ | |
| | c.5465T>A | ■ | ■ | ■ | |
| | c.5880G>A | ■ | ■ | ■ | |
| ATR | c.7274G>A | ■ | ■ | | |
| | c.1953G>A | | | | ■ |
| | c.2211C>T | | | | ■ |
| | c.2550A>C | | | | ■ |
| AXIN1 | c.1284G>A | ■ | ■ | | |
| | c.1827T>C | ■ | ■ | | |
| | c.1549G>A | ■ | ■ | | |
| | c.4563A>G | | | | ■ |
| | c.6668C>T | | | | ■ |
| | c.2742A>G | | | | ■ |
| AXIN2 | c.331= | | | | ■ |
| | c.402T>C | | | | ■ |
| BAP1 | c.501G>A | ■ | | | |
| BLM | c.3102G>A | ■ | | | |
| | c.3531C>A | ■ | | | |
| | c.3945C>T | ■ | | | |
| BRAF | c.*1363G>A | | | | ■ |
| | c.*1483A>G | | | | ■ |
| | c.*813T>C | | | | ■ |
| | c.*922G>C | | | | ■ |
| | c.3324A>G | | | | ■ |
| | c.5094C>T | | | | ■ |
| | c.6555C>T | | | | ■ |
| | c.75+60C>T | | | | ■ |
| BRCA1 | c.3113A>G | ■ | ■ | ■ | |
| | c.3119G>A | ■ | ■ | ■ | |

| | | | | | |
|---|---|---|---|---|---|
| | c.3548A>G | | | | |
| | c.4308T>C | | | | |
| | c.4837A>G | | | | |
| | c.5075-237C>A | | | | |
| | c.18C>T | | | | |
| | c.2783-181G>A | | | | |
| | c.2989A>G | | | | |
| | c.3183T>A | | | | |
| | c.320-2491 A>T | | | | |
| | c.320-4481 A>G | | | | |
| | c.3333C>T | | | | |
| CD276 | c.540+7405G>A | | | | |
| | c.58+1134C>T | | | | |
| CHD2 | c.1389A>G | | | | |
| | c.1638_1650delinsTGGGCAGGATGCA | | | | |
| | c.1782A>G | | | | |
| | c.783C>T | | | | |
| | c.2049A>G | | | | |
| | c.2718A>G | | | | |
| | c.3564C>T | | | | |
| | c.5416A>C | | | | |
| CIC | c.3052G>A | | | | |
| | c.6018T>C | | | | |
| | c.7260C>T | | | | |
| CRLF2 | c.33C>G | | | | |
| E2F3 | c.1000-110G>A | | | | |
| | c.1136-736A>C | | | | |
| | c.2169+1275A>G | | | | |
| | c.2169+1465T>C | | | | |
| | c.2169+54A>G | | | | |
| | c.2169+934G>T | | | | |
| | c.330A>T | | | | |
| | c.5061+1034A>G | | | | |
| | c.5062-523T>C | | | | |
| EGFR | c.1839C>T c.1880+55C>T | | | | |
| | c.1881-1267C>A | | | | |
| | c.1881-1585A>G | | | | |
| | c.1881-2094A>G | | | | |
| | c.1881-404T>G | | | | |
| | c.1881-721G>A | | | | |
| | c.1887T>A | | | | |
| | c.2283+125T>C | | | | |
| | c.2283+1296C>T | | | | |
| | c.2283+1901G>A | | | | |
| | c.2283+2225C>T | | | | |
| | c.2283+269T>C | | | | |
| | c.2283+2994A>G | | | | |
| | c.274T>C | | | | |
| | c.2868T>C | | | | |
| | c.2983C>T | | | | |
| | c.3014G>A | | | | |

| | | | | | |
|---|---|---|---|---|---|
| | c.3127T>C | | | | ■ |
| | c.489C>T | | | | ■ |
| | c.563G>A | | | | ■ |
| | c.622-248G>A | | | | ■ |
| | c.686T>C | | | | ■ |
| | c.738C>T | | | | ■ |
| ESR1 | c.1096+21499_1096+21500 | ■ | ■ | ■ | |
| | c.1236-18935G>A | ■ | ■ | ■ | |
| | c.236-12072G>A | | | | ■ |
| | c.236-12121T>C | | | | ■ |
| | c.236-12350A>G | | | | ■ |
| | c.236-5004G>A | | | | ■ |
| | c.236-6047A>T | | | | ■ |
| | c.236-9376C>G | | | | ■ |
| | c.236-9652G>T | | | | ■ |
| ETV1 | c.182-3581A>G | | | ■ | |
| | c.182-3990C>T | | | ■ | |
| | c.182-4016G>C | | | ■ | |
| | c.182-4229 182-4228delinsGC | | | ■ | |
| | c.235+113A>G | | | ■ | |
| | c.235+11542C>T | | | ■ | |
| FLI1 | c.203G>T | | ■ | ■ | |
| GRM3 | c.879C>T | ■ | ■ | | |
| H3F3C | c.*2T>G | ■ | ■ | | |
| KRAS | c.*512T>C | ■ | ■ | | |
| | c.-12+2376_-12+2391dup | ■ | ■ | | |
| | c.2049A>G | | | | ■ |
| | c.2225G>C | | | | ■ |
| | c.2547= | | | | ■ |
| | c.2629A>T | | | | ■ |
| | c.2718A>G | | | | ■ |
| | c.387G>A | | | | ■ |
| | c.3906T>C | | | | ■ |
| | c.451-5617G>A | ■ | ■ | ■ | |
| | c.451-7404A>C | ■ | ■ | ■ | |
| MAP3K1 | c.-6-2208A>C | | | | ■ |
| | c.-6-7161G>A | | | | ■ |
| | c.951G>T | | | | ■ |
| MSH6 | c.1189+2922T>C | | | | ■ |
| | c.1189+699C>T | | | | ■ |
| | c.1190-1609C>T | | | | ■ |
| | c.186C>A | ■ | ■ | | |
| | c.276A>G | ■ | ■ | | |
| | c.540T>C | ■ | ■ | | |
| | c.956C>T | ■ | ■ | | |
| PAX3 | c.1173+3075T>G | ■ | ■ | | |
| | c.1173+4667C>T | ■ | ■ | | |
| | c.1173+5515C>T | ■ | ■ | | |
| | c.1173+8919A>C | ■ | ■ | | |
| | c.1174-10G>C | ■ | ■ | | |
| | c.2412A>G | | | | ■ |

| | | | | | |
|---|---|---|---|---|---|
| | c.67-11872T>C | | | | |
| | c.804T>C | | | | |
| | c.-8-11265C>T | | | | |
| | c.-8-12925A>G | | | | |
| | c.-8-16713T>C | | | | |
| | c.-8-18084G>T | | | | |
| | c.-8-18602A>G | | | | |
| | c.-8-18729C>T | | | | |
| | c.-8-23811A>G | | | | |
| | c.-8-24615G>A | | | | |
| | c.-8-24969C>T | | | | |
| | c.-8-25230del | | | | |
| | c.-8-9088G>A | | | | |
| PDGFRA | c.1701 A>G | | | | |
| | c.1787-269G>A | | | | |
| | c.3222T>C | | | | |
| PTEN | c.80-3023C>T | | | | |
| RET | c.2136+182G>A | | | | |
| | c.2136+271T>C | | | | |
| | c.2136+374C>T | | | | |
| | c.2137-167T>C | | | | |
| | c.2137-324A>G | | | | |
| | c.2284+430C>T | | | | |
| | c.2284+47C>T | | | | |
| | c.450+4044C>T | | | | |
| | c.463+1398C>G | | | | |
| | c.463+2405G>T | | | | |
| | c.463+3246A>G | | | | |
| | c.463+4241C>T | | | | |
| | c.463+49G>A | | | | |
| | c.463+5025G>A | | | | |
| | c.463+5273 | | | | |
| | 463+5277delinsAG | | | | |
| | c.463+5558G>C | | | | |
| | c.463+6186T>C | | | | |
| | c.463+6306A>G | | | | |
| | c.463+6694A>G | | | | |
| | c.463+6797G>A | | | | |
| | c.463+7077G>A | | | | |
| | c.463+7117_463+7131delinsATTATTTGGTTGTTTC | | | | |
| | c.463+848G>C | | | | |
| ROS1 | c.5231-1578A>G | | | | |
| | c.5231-191C>T | | | | |
| | c.5231-2516A>G | | | | |
| | c.5231-2735T>C | | | | |
| | c.5231-3291C>T | | | | |
| | c.5231-3311T>C | | | | |
| | c.5231-415C>T | | | | |
| | c.5231-735T>C | | | | |
| | c.5348+1319G>A | | | | |
| | c.5348+361T>A | | | | |

| | | | | | |
|---|---|---|---|---|---|
| | c.5624-1461G>T | | | ■ | |
| | c.5624-876A>G | | | ■ | |
| | c.5759+351 del | | | ■ | |
| | c.5759+468A>C | | | ■ | |
| | c.5759+603A>C | | | ■ | |
| | c.5760-137G>A | | | ■ | |
| | c.5760-281C>T | | | ■ | |
| | c.5760-53G>T | | | ■ | |
| | c.5922+434G>C | | | ■ | |
| TP53 | c.-28-1184T>C | ■ | ■ | | |
| | c.-28-3073G>C | ■ | ■ | | |
| | c.-28-3143C>T | ■ | ■ | | |
| | c.-29+2135T>C | ■ | ■ | | |
| | c.-29+2225T>A | ■ | ■ | | |
| ZFHX3 | c.4446A>T | ■ | ■ | ■ | |
| | c.8823A>G | ■ | ■ | ■ | |

**Table S2**. Complete mutations set found in subject B, two premolar teeth analyzed at different PMI/ADDs, with mutations found at specific PMI/ADDs. Case 10: LRSP at PMI 17; case 2: URSP at PMI 30. Coloured boxes represent the occurrence of mutations; clear boxes represent the absence of mutations.

| **PMI (Days)** | | **17** | **30** |
|---|---|---|---|
| **ADD (°C)** | | **440.9** | **738.1** |
| **GENES** | **MUTATIONS** | | |
| ABRAXAS1 | c.1117G>A | | ■ |
| ADGRA2 | c.3660C>T | | ■ |
| AKT2 | c.-84-5059-84-5052 | | ■ |
| AKT3 | c.*3694G>A | ■ | |
| | c.*3909T>C | ■ | |
| | c.*3731C>T | | ■ |
| ALK | c.1500A>G | | ■ |
| | c.3067+365C>T | | ■ |
| | c.3068-329G>A | | ■ |
| | c.3068-415G>C | | ■ |
| | c.3359+24G>C | | ■ |
| | c.3375C>A | | ■ |
| | c.4472A>G | | ■ |
| | c.3068-574C>A | ■ | |
| | c.4338C>T | ■ | |
| AMER1 | c.135C>T | | ■ |
| ATR | c.7274G>A | | ■ |
| APC | c.7504G>A | ■ | |
| AR | c.1616+10462G>A | ■ | |
| AURKB | c.893T>C | ■ | |
| AXIN1 | c.1045C>T | ■ | |
| | c.1284G>A | ■ | |
| | c.1549G>A | ■ | |
| | c.1827T>C | ■ | |

| | | | |
|---|---|---|---|
| BARD1 | c.70C>T | | |
| BCL10 | c.13G>T | | |
| BCL2L11 | c.394+3056G>C | | |
| | c.394+3849C>T | | |
| BCL2L2 | c.128A>G | | |
| BCL6 | c.492G>T | | |
| BCR | c.2387A>G | | |
| | c.2708-193G>A | | |
| | c.2782+182 | | |
| BLM | c.1046G>A | | |
| BMPR1A | c.1140C>T | | |
| | c.1140+2755_1140+2776 | | |
| | c.1140+2850G>A | | |
| | c.1140+757A>G | | |
| BRAF | c.1140+2430C>T | | |
| | c.1140+2755_1140+2776 | | |
| | c.1140+2850G>A | | |
| | c.1140+757A>G | | |
| BRCA1 | c.-19-216A>G | | |
| | c.2311T>C | | |
| | c.2612C>T | | |
| | c.3119G>A | | |
| | c.3548A>G | | |
| | c.4308T>C | | |
| | c.4837A>G | | |
| | c.5075-237C>A | | |
| | c.134+1967T>C | | |
| | c.134+2983_134+3004delinsAAACCCCTACTGATGAA | | |
| | c.-19-115T>C | | |
| | c.3113A>G | | |
| | c.399T>C | | |
| CCND1 | c.723+571 | | |
| | c.723G>A | | |
| CCND2 | c.493C>T | | |
| CCNE1 | c.1215C>T | | |
| | c.399T>C | | |
| DOT1L | c.4156G>A | | |
| EGFR | c.2284-1652C>T | | |
| | c.2284-199T>C | | |
| | c.2284-2766G>A | | |
| | c.2284-498G>A | | |
| | c.2284-60T>C | | |
| | c.2284-755A>G | | |
| | c.89-25201A>G | | |
| | c.1881-781C>T | | |
| | c.747+548C>T | | |
| | c.748-235C>T | | |
| | c.1881-721G>A | | |
| EML4 | c.*1941A>G | | |
| ETV1 | c.181+1867T>A | | |
| | c.181+1883G>A | | |

| | | | |
|---|---|---|---|
| | c.181+536G>A | | ■ |
| | c.181+623C>T | | ■ |
| | c.181+735G>T | | ■ |
| | c.181+789T>C | | ■ |
| | c.181+852A>C | | ■ |
| | c.181+894A>G | | ■ |
| | c.181+946T>C | | ■ |
| ETV6 | c.463+7490C>A | ■ | |
| | c.463+7929G>A | ■ | |
| | c.463+848G>C | ■ | |
| | c.464-6175G>T | ■ | |
| | c.464-6269C>T | ■ | |
| FAT1 | c.8523C>T | ■ | |
| FLI1 | c.203G>T | ■ | |
| | c.942C>G | | ■ |
| HNF1A | c.1375C>T | | ■ |
| | c.1460G>A | | ■ |
| KAT6A | c.401T>C | | ■ |
| | c.4455C>T | | ■ |
| KDM5A | c.2594T>C | | ■ |
| | c.4149C>T | | ■ |
| ROS1 | c.5624-1340T>C | ■ | |
| RPS6KB2 | c.1259C>T | | ■ |
| | c.807C>T | | ■ |
| RPTOR | c.1269C>T | | ■ |
| TNFRSF14 | c.50A>G | ■ | |
| TP53 | c.1100+154C>G | ■ | |
| ZFHX3 | c.1212C>T | ■ | |
| | c.1282A>C | ■ | |
| | c.1602C>T | ■ | |

**Table S3.** Mutations found in teeth extracted from different subjects and analyzed at same PMI/ADDs. Column 2 reports the results obtained from a sample analyzed at PMI 8 in the 2022 pilot study and compared with the results obtained from sample 8 (Column 1) from this study. Columns 3 to 11 compare the samples analyzed in this study. Coloured boxes represent the occurrence of mutations; clear boxes represent the absence of mutations.

| PMI (Days) | | 8 | 8 | 16 | 16 | 17 | 23 | 28 | 30 | 30 | 35 | 35 |
|---|---|---|---|---|---|---|---|---|---|---|---|---|
| ADD (°C) | | 157,4 | 183,1 | 369,2 | 388,2 | 440,9 | 551,2 | 678,3 | 581,5 | 738,1 | 753,8 | 753,8 |
| GENES | MUTATIONS | | | | | | | | | | | |
| ABRAXAS1 | c.1117G>A | | | | | | | | | ■ | ■ | ■ |
| AKT3 | c.*3909T>C | ■ | ■ | ■ | ■ | ■ | ■ | ■ | | | | |
| ALK | c.3067+365C>T | ■ | | | | | | | | ■ | ■ | ■ |
| | c.3068-415G C | ■ | | | | | | | | ■ | ■ | ■ |
| | c.3068-574C>A | | | ■ | ■ | ■ | ■ | ■ | ■ | | | |
| | c.3359+24G>C | | | | | | | | | ■ | ■ | ■ |
| | c.3375C>A | | | | | | | | | ■ | ■ | ■ |
| | c.4338C>T | | | ■ | ■ | ■ | ■ | ■ | | | | |
| | c.4472A>G | | | | | | | | | ■ | ■ | ■ |
| AMER1 | c.135C>T | | | | | | | | | ■ | ■ | ■ |
| ANKRD26 | c.4204C>T | | | | | | | | | ■ | ■ | ■ |
| ATR | c.7274G>A | ■ | ■ | ■ | ■ | | | | | | | |
| AXIN1 | c.1284G>A | | | ■ | ■ | ■ | ■ | | | | | |
| | c.1549G>A | | | ■ | ■ | ■ | ■ | | | | | |
| | c.1827T>C | | | ■ | ■ | ■ | ■ | | | | | |
| BAP1 | c.501G>A | ■ | ■ | ■ | ■ | | | | | | | |
| BCL2L11 | c.394+3056G>C | | | | | | | | | ■ | ■ | ■ |
| | c.394+3849C>T | | | | | | | | | ■ | ■ | ■ |

| | | | | | | | | | | | | |
|---|---|---|---|---|---|---|---|---|---|---|---|---|
| BCL2L2 | c.128A>G | | | | | | | | | | | |
| BCL6 | c.492G>T | | | | | | | | | | | |
| BLM | c.1046G>A | | | | | | | | | | | |
| | c.3102G>A | | | | | | | | | | | |
| | c.3531C>A | | | | | | | | | | | |
| | c.3945C T | | | | | | | | | | | |
| BMPR1A | c.1140C>T | | | | | | | | | | | |
| BRAF | c.1140+757A>G | | | | | | | | | | | |
| | c.1141-1305T>C | | | | | | | | | | | |
| BRCA1 | c.134+1967T>C | | | | | | | | | | | |
| | c.134+2983_134+3004delinsAAACCC CTACTGATGAA | | | | | | | | | | | |
| | c.-19-115T>C | | | | | | | | | | | |
| | c.-19-216A>G | | | | | | | | | | | |
| | c.3113A>G | | | | | | | | | | | |
| | c.3119G>A | | | | | | | | | | | |
| | c.3548A>G | | | | | | | | | | | |
| | c.4308T>C | | | | | | | | | | | |
| | c.4837A>G | | | | | | | | | | | |
| | c.5075-237C>A | | | | | | | | | | | |
| CCND1 | c.723+571 | | | | | | | | | | | |
| | c.723G>A | | | | | | | | | | | |
| EGFR | c.1881-721G>A | | | | | | | | | | | |
| | c.1881-781C>T | | | | | | | | | | | |
| | c.747+548C>T | | | | | | | | | | | |
| | c.748-235C>T | | | | | | | | | | | |
| | c.89-25201A>G | | | | | | | | | | | |
| EML4 | c.*1941A>G | | | | | | | | | | | |
| ERBB2 | c.2086-29G>A | | | | | | | | | | | |
| | c.2273delA | | | | | | | | | | | |
| FAT1 | c.8523C>T | | | | | | | | | | | |
| FGFR3 | c.2393delC | | | | | | | | | | | |
| | c.840C>T | | | | | | | | | | | |
| | c.843A>C | | | | | | | | | | | |
| FLI1 | c.203G>T | | | | | | | | | | | |
| | c.942C>G | | | | | | | | | | | |
| HNF1A | c.1375C>T | | | | | | | | | | | |
| | c.1460G>A | | | | | | | | | | | |
| KAT6A | c.401T>C | | | | | | | | | | | |
| | c.4455C>T | | | | | | | | | | | |
| KDM5A | c.2594T>C | | | | | | | | | | | |
| | c.4149C>T | | | | | | | | | | | |
| PDGFRA | c.2003-11delT | | | | | | | | | | | |
| PTEN | c.802-18delC | | | | | | | | | | | |
| | c.802-19dupT | | | | | | | | | | | |
| | c.802-23C>T | | | | | | | | | | | |
| | c.802-29C>T | | | | | | | | | | | |
| | c.80-3023C>T | | | | | | | | | | | |
| ROS1 | c.5624-1340T>C | | | | | | | | | | | |
| TNFRSF14 | c.50A>G | | | | | | | | | | | |
